# Kynurenic Acid Acts as a Signaling Molecule Regulating Energy Expenditure and Is Closely Associated With Metabolic Diseases

**DOI:** 10.3389/fendo.2022.847611

**Published:** 2022-02-24

**Authors:** Delong Zhen, Junjun Liu, Xu Dong Zhang, Zehua Song

**Affiliations:** ^1^ Translational Research Institute, Henan Provincial People’s Hospital and People’s Hospital of Zhengzhou University, Academy of Medical Science, Zhengzhou University, Zhengzhou, China; ^2^ Shandong Institute of Endocrine and Metabolic Diseases, Shandong First Medical University & Shandong Academy of Medical Sciences, Jinan, China; ^3^ ENNOVA Institute of Life Science and Technology, ENN Group, Langfang, China

**Keywords:** kynurenic acid, inflammation, physical exercise, perinatal nutrition, metabolic diseases

## Abstract

Kynurenic acid (KYNA) is an important bio-active product of tryptophan metabolism. In addition to its well-known neuroprotective effects on mental health disorders, it has been proposed as a bio-marker for such metabolic diseases as atherosclerosis and diabetes. Emerging evidence suggests that KYNA acts as a signaling molecule controlling the networks involved in the balance of energy store and expenditure through GPR35 and AMPK signaling pathway. KYNA plays an important role in the pathogenesis and development of several endocrine and metabolic diseases. Exercise training promotes KYNA production in skeletal muscles and increases thermogenesis in the long term and limits weight gain, insulin resistance and inflammation. Additionally, KYNA is also present in breast milk and may act as an anti-obesity agent in infants. Although we are far from fully understanding the role of KYNA in our body, administration of KYNA, enzyme inhibitors or metabolites may serve as a potential therapeutic strategy for treating metabolic diseases. The present review provides a perspective on the current knowledge regarding the biological effects of KYNA in metabolic diseases and perinatal nutrition.

## Introduction

Kynurenic acid (KYNA) is one of the metabolites of tryptophan catabolism formed *via* the kynurenine pathway. It is first known for its neuro-protective effect as it is the only known broad-spectrum endogenous antagonist for ionotropic glutamate receptors. As such, a large number of studies have been carried out to investigate the role of KYNA in the physio-pathology of central nervous system (CNS) such as depression, Alzheimer’s diseases and schizophrenia in the past two decades. In addition to such neuronal contributions, KYNA can also be found with higher concentration in urine, pancreatic mucus, serum and breast milk than in cerebrospinal fluid (1). It has been subsequently found to be involved in immune (2) and digestive system (1) in the periphery. Increasing reports have concentrated on the role of KYNA outside the CNS.

More recently, evidence suggests that physical exercise may also influence this pathway and KYNA has emerged as a signaling molecule for energy homeostasis in peripheral tissues. Some metabolomic and epidemiological studies have proposed that KYNA can serve as an early bio-marker for diabetes and some other metabolic diseases (3, 4). Furthermore, in some animal studies, KYNA has been considered as a significant protector against such metabolic diseases as obesity and nonalcoholic fatty liver disease (NAFLD) (5, 6). This essay summarizes recent advances of how KYNA is involved in the regulation of energy homeostasis of peripheral tissue and its potential role in the onset and progression of such metabolic diseases as obesity and diabetes.

## The Endogenous Production of KYNA in Peripheral Tissues

The endogenous production of KYNA in human brain has been well studied over the past few decades because of its neuroprotective effects. However, increasing evidence showed that KYNA can also be found, even in higher concentration, in many body fluids in humans such serum (7), saliva (8), bile (9) and breast milk (10). In humans and rodents, the production of KYNA has been described in a number of peripheral tissues such as muscle (11), liver (12), kidney (12), pancreas (9, 13), endothelial cells (14) and immune cells (15) under physiological conditions. KYNA is produced mainly through the side branch of tryptophan/kynurenine pathway. Approximately 95% of tryptophan is metabolized through kynurenine pathway, and about 0.3% is converted to KYNA (10 µmol/day) (16).

In this catabolic pathway, the first step is catalyzed by the enzymes indol-2,3-dioxygenase (IDO) and tryptophan-2,3-dioxygenase (TDO) to generate N-formyl-L-kynurenine, an unstable compound which is rapidly converted to L-kynurenine (L-KYN) by ubiquitous aryl formamidase (AFMID). TDO is mainly expressed in the liver and has a *K_m_* value of 190 µM for TRP, which ensures that TDO is active to convert TRP to L-KYN at higher than physiological concentrations (about 80 µM) (17, 18). Another limiting enzyme, IDO1, is expressed under the induction of proinflammatory cytokines (19). IDO1 has a *K_m_* value of 20 µM for tryptophan, which is much lower than TDO (17). IDO2, a newly discovered enzyme, has some homology to IDO1, but its *K_m_* value for TRP is much higher than that of IDO1 and TDO, and has little effect on the production of TRP downstream metabolites (20). After this step, L-KYN can be either converted to nicotinamide by a series of reactions or enter into a side branch to produce KYNA. KYNA is supposed to be a final catabolic product of this side branch of kynurenine pathway because no further metabolite is reported in mammals (but can be further catabolized by intestinal flora).

The KYNA branch of the kynurenine pathway is mainly regulated by the activity of kynurenine aminotransferase (KYAT, EC 2.6.1.7), whose abbreviation is recently updated from KAT to KYAT. Four different isozymes of KYAT (KYAT1-4) are identified in mammalian cells. They are all members of the pyridoxal-5’-phosphate-dependent enzyme family and require an α-ketoacid as the amino group acceptors.

KYAT1 catalyzes the transamination of L-KYN to form KYNA and its *K_m_* for L-KYN is around 4.7 mM (21). It should be noted that KYAT1 possesses broad amino acid specificity and also catalyzes the transamination such as glutamine to α-ketoglutaramate, thus it is also known as glutamine transaminase K (GTK, EC 2.6.1.15). Leucine, glutamate, methionine also seem to be the preferred substrates of human KYAT1 (21). But, to date, its most biologically significant product is KYNA. KYAT1 also possesses cysteine S-conjugate beta-lyases (CCBL1) activity and catalyzes the beta-elimination of cysteine S-conjugates to generate pyruvate and thioacylating fragments. This activity plays an important role in the bio-activation of cysteine S-conjugates found in garlic and onion (22) and in the toxification of some toxins like 5-S-l-cysteinyldopamine. Competitive inhibition test suggests KYAT1 has two active sites, one for KYAT and the other for GTK. The active sites of KYAT and CCBL1 may be the same (23). KYAT1 is both cytosolic and mitochondrial because it exists two different mRNA variants coding for proteins with and without mitochondria targeting sequence. In rats, KYAT1 mRNA can be detected in most of tissues such as small intestine, pancreas, lung, liver, heart, kidney, brain, muscle, testis, ovary (23, 24). In brain, the activity of KYAT1 is critical to the formation of KYNA, its activity is associated with schizophrenia (25). A missense mutation in KYAT1 was identified in spontaneously hypertensive rats. This mutation led to abnormally low KYNA levels in the area of central nervous system that controls blood pressure (26). Although the role of KYAT1 in brain has mostly been discussed, its expression is much higher in livers and kidneys than in brains (27). Its role in peripheral tissues did not receive much attention until the discovery of the immunomodulator and metabolic effect of KYNA.

KYAT2, the second isoform of the KYAT family, also possesses a broad-spectrum transamination activity with no S-conjugate beta-lyases activity. It is also known as alpha-aminoadipate aminotransferase (AADAT). Similar to KYAT1, its *K_m_* for L-KYN is around 4.7 mM (28). KYAT2 can be detected in most tissues, but it is not detectable in murine skeletal muscle (11). KYAT2 appears to play a more important role in rat’s brain because KYAT2 is highly expressed in astrocytes than other KYATs and KYNA is reported to be predominantly generated by it in the brain (29, 30).

Among the four KYATs, KYAT3 shares similar sequence and expression pattern to KYAT1 and is also known as glutamine transaminase L (GTL) and CCBL2 (EC 4.4.1.13). Likewise, KYAT3 is a multifunctional aminotransferase and catalyzes glutamine, methionine, phenylalanine, tyrosine and cysteine as transamination subtract, although it displayed no activity toward leucine (31). Its expression is much higher in kidney, liver and neuroendocrine tissues than in brain (27, 32).

KYAT4 is the last discovered KYAT. In fact, it is better known as mitochondrial glutamic-oxaloacetictransaminase 2 (EC 2.6.1.1), an essential player in the malate-aspartate shuttle in mitochondria and in the synthesis of glutamate (33). It is highly expressed in most tissues and organs because malate-aspartate shuttle is a general feature of cells with functional mitochondria, except for white adipose tissue (34).

Kynurenine 3-monooxygenase (KMO; EC 1.14.13.9) is an important regulator of KYNA synthesis as it is a kynurenine-consuming enzyme competing with KYAT for substrate. KMO is an NADPH-dependent flavin monooxygenase located in the outer membrane of mitochondria. It catalyzes the conversion of kynurenine to 3-hydroxykynurenine, a cytotoxic metabolite involved in the generation of ROS and activation of inflammatory response. KMO is widely expressed in our body, for it has been discovered in liver, kidney, pancreas, brain, macrophages, and monocytes (3, 13, 35, 36), with the highest KMO levels found in liver and kidney. Moreover, the activity of KMO in liver and kidney decreases significantly with aging (37).

KMO possesses higher affinity to kynurenine (7-14 μM for human KMO (38, 39), 15-16 μM for rodent (40) than all four types of KYATs. KMO inhibition by pharmacological inhibitor significantly increases KYNA levels (41). Also, in KMO knockout mice, the level of KYN and KYNA was significantly increased in the periphery (42). The characteristics of enzymes related to KYNA metabolism are summarized in **Table 1**.

**Table 1:** The characteristics of enzyme related to KYNA metabolism.

Enzyme	*K_m_*	Substrate	References
TPH1	8 μM	Trp	(43)
TPH2	41.3 μM	Trp	(44)
IDO1	20 μM	Trp	(45)
TDO2	190 μM	Trp	(17)
KMO	7-16 μM	KYN	(38–40)
Kase	493 μM	KYN	(46)
KYAT1	4700 μM	KYN	(21)
KYAT2	4700 µM	KYN	(28)
KYAT3	1500 µM	KYN	(31)

Under some physiological or pathological conditions, KYNA can be produced from indole-3-Pyruvic Acid or from kynurenine by scavenging free radicals. These pathways represent alternative routes of KYNA production. Although the contributions of these alternative routes remain unclear, these could be very important in such metabolic diseases as obesity and diabetes, because these diseases share common factors such as oxidative stress and inflammation. This information has been reviewed by Ramos-Chávez et al. (47).

## The Transport of KYNA

KYNA is able to cross the plasma membrane through organic anion transporters 1 and 3 (OAT1/SLC22A6 and OAT3/SLC22A8) (48). The proximal tubule of the kidney, where OATs are found, is not simply for renal elimination of KYNA and it also senses tryptophan metabolites levels and responds to changes in their intracellular abundance (49). It remains to be further investigated whether there exists an exocytosis gated KYNA secretion.

While KYNA does not easily cross the blood-brain barrier, KYNA synthesized by brain cannot be directly exported to the periphery and *vice versa* (50). However, since its precursor L-KYN can cross the blood-brain barrier (51), KYNA can be synthesized by KYAT *in situ* using transported L-KYN in central nervous system under certain conditions (51). Moreover, a recent study showed that in *Caenorhabditis elegans*, an ortholog of the human LAT1 transporter, AAT-1, imports L-KYN into sites of KYNA production (52). Another study showed that five amino acids, including leucine, isoleucine, methionine, phenylalanine and tyrosine, act as LAT substrates and inhibit brain KYNA synthesis by blocking L-KYN transport (53). Similar to this transport mechanism in brain, the uptake of KYNA in T cells can be mediated by the uptake of L-KYN *via* L-amino acid transporter SLC7A5 (54).

## Links Between KYNA and Common Metabolic Diseases

### Inflammation, the First Link Between KYNA and Metabolic Diseases

The production of KYNA is directly correlated to inflammation as KYNA acts as an important immune regulated during inflammation response. KYNA inhibits TNF-α at transcriptional level and suppresses the secretion of TNF-α in mononuclear cells and in CD14^+^ monocytes (55). Oral administration of KYNA decreases the activity of the peripheral blood leukocytes in mice (56).

The immune response signaling pathway and metabolic regulation signaling pathway, especially insulin signaling pathway, are highly integrated, because organism would need to redistribute its energy resources during the activation of immune response (57). Chronic inflammation is activated in overweight individuals as a consequence of adipose expansion. Recent insights suggest that it may play an indispensable role in the over-nutrition induced insulin resistance (58). During the past two decades, it became clear that nutrient excess and activation of the innate immune system are highly associated in most organs such as adipose tissues, liver, gut, muscle, and islets (58). Low-grade chronic inflammation or metabolically triggered inflammation is considered as a fundamental characteristic of metabolic diseases particularly in the context of obesity and type 2 diabetes. Targeting inflammation has been suggested as an important strategy to prevent and control these diseases (58).

Numbers of studies suggest that KYNA is produced during inflammation and it has been shown to mediate various immunomodulatory effects under inflammatory conditions (2). Since inflammation is one of the main factors in many metabolic diseases, it can be foreseen that KYNA may also play an important regulatory role in the metabolic diseases.

Increased tryptophan/kynurenine metabolite levels are frequently observed in overweight individuals (3). An increased serum KYNA level can be found in Zucker fatty rats (59), and in HFD fed LDL receptor knockout mice (60). Clinically, serum KYNA has been found positively correlated with BMI in overweight individuals (3, 61). The elevation of serum KYNA concentrations is closely associated with the activation of immune cells as increased IDO1 activity in macrophages and increased serum KYNA levels have been reported in obese animal models. The increased KYNA levels may result from an up-regulated biosynthesis in the omental adipose tissue (but not in subcutaneous adipose tissue), as the expression levels of IDO1, KYAT1 and KYAT3 were significantly higher in overweight individuals than in lean individuals (3). Furthermore, the activation of KYNA production was not restrained in resident immune cells of adipose tissue as the increased expression of IDO1, KYAT2 and KYAT3 can also be found in adipocytes. This up-regulation may be due to the increased production of pro-inflammatory cytokines from resident immune cells since adipocyte does not express KMO (3). However, in another study, Pyun et al. found a negative correlation between serum KYNA levels and BMI (6). These controversial observations may be due to the different criteria. Another possible explanation to the controversy is that the KYNA determination method used in these studies are different: the last one used an ELISA kit to determinate serum KYNA levels, while the others used the HPLC-MS/MS method. It needs to be further verified whether these divergent results are due to some immeasurable confounding factors.

Liver seems to be another important source of serum KYNA in overweight individuals as TDO and KYATs are highly expressed in this organ (17, 18). Moreover, overweight is frequently associated with a low-grade chronic inflammation with an induction of IDO1 in liver (62). Furthermore, both of the liver and adipose tissue have a closed crosstalk between resident immune cells (Kupffer cells or macrophages) and metabolic cells (adipocytes or hepatocytes). However, to the best of our knowledge, neither the production of KYNA nor the regulation of IDO, TDO and KYATs in hepatocytes under metabolic challenge have been reported.

### Physical Exercise, the Second Link Between KYNA and Metabolic Diseases

Physical exercise has been described as a promising non-pharmacological treatment for overweight and some other metabolic diseases (63). In addition to its role in energy expenditure regulation, skeletal muscle is increasingly considered as one of the largest endocrine organs in our body. It secretes a variety of myokines and bioactive metabolites, which exerts important effects on the regulation of metabolism and inflammation. KYNA can also be synthesized by skeletal muscle and its production is closely correlated to the physical exercise in both human and mouse (11, 64).

All the four KYATs are expressed in skeletal muscle, but KYAT isoforms display fiber-type specific expression. KYAT1, KYAT3 and KYAT4 are more abundant in oxidative type I than glycolytic type II fiber (65). Accordingly, an increased serum KYNA level has been found in individuals after endurance exercise (64). Within the first hour after aerobic exercise, there is an increase in plasmatic KYNA and this effect lasts for 2 hours after exercise. In contrast, high-intensity eccentric exercise did not lead to increased plasmatic KYNA concentration (64). Regarding the effect of long-term exercise on KYNA, a recent study of 4-week physical exercises on human found that physical exercises promote an increase in the amount of KYNA in sweat on day 14. The KYNA level returned to baseline on day 28 (66). Additionally, inhibition of KYATs reduces myotube oxidative capacity and exercise performance in mice (67).

Physical exercise induces peroxisome-proliferator activated receptor γ coactivator 1α (PGC-1α) expression in skeletal muscle (11). PGC1-1α coordinates the expression of several genes involved in the adaptive energy metabolism and fatigue-resistance such as mitochondrial biogenesis and fatty acid oxidation. Recently, the canonical and longest transcript variant of PGC-1α, PGC-1α1, is reported to up-regulate KYAT2 and KYAT4 expression (11). Such mechanism in skeletal muscle during physical exercise may be primarily aimed at enhancing the malate-aspartate shuttle as both KYATs are important enzymes in the malate-aspartate shuttle (67). Consequently, this mechanism shifts the kynurenine metabolism to KYNA production.

This exercise-induced KYNA production is originally described as the crosstalk between skeletal muscle and the brain to elucidate the effectiveness of exercise in reducing depressive symptoms. A recent study by Agudelo et al. demonstrated that KYNA increases energy utilization by activating G-protein-coupled receptor 35 (GPR35), which stimulates lipid metabolism, thermogenic, and anti-inflammatory gene expression in adipose tissue (68). Also, GPR35 agonists was reported to suppress high fat diet-induced fatty liver development (5). These data uncovered that skeletal muscle derived from KYNA may be a potential regulator of energy homeostasis and a coordinator of exercise-induced adaptations in other organs including liver, adipose tissue and brain.

However, it should be noted that physical exercise induces strong and transit increases in KYNA levels while inflammation leads to mild and sustained increases in KYNA levels.

### Perinatal Nutrition, The Third Link Between KYNA and Metabolic Diseases

Epidemiological and experimental data have suggested that perinatal nutrition has a significant role in the development of lifelong metabolic disorders (69). KYNA may also act as a link between perinatal offspring and mother. KYNA can pass through the placenta into the fetus (70). However, placental and fetal KYNA were not affected by placental infusion of L-KYN in mice (70). Also, under physiological conditions, KYNA was higher in the liver and brain of mouse’s fetuses than in the placenta, and KYNA in the fetus was not affected during oral maternal administration of KYNA. It can be hypothesized that maternal KYNA cannot affect fetus through placenta (70, 71).

Although maternal KYNA cannot directly affect the fetus, some studies found the KYNA content in breast milk gradually increases in different lactation periods (10). Epidemiological studies showed a slower body weight gain in naturally fed newborns compared to artificially fed ones (72). Although the formula milk powder for infants in different periods are different, studies have found that KYNA content in formula milk powder is much lower than that in breast milk (10). Rats postnatally exposed to KYNA supplementation were observed to have a significant reduction of body weight gain, but no changes in total body surface and bone mineral density. The rat offspring supplemented with KYNA presents a lower mass gain during the first 21 days of life, which indicates that KYNA may act as an anti-obese agent (10).

Another potential mechanism is that perinatal KYNA may be protective against overweight by modulating the gut microbiota. Formula feeding appears to promote the microbiota associated with overweight (73), while KYNA stimulates the growth of certain probiotics (74). It still needs to be further explored whether the presence of KYNA in breast milk acts as a modulator of gut microbiota.

## Potential Mechanism of KYNA Involved in Common Metabolic Diseases

### Glutamate Receptors

KYNA is well-known for its role as an endogenous N-methyl-D-aspartate receptor (NMDAR) antagonist in the brain. In the periphery, the expression of functional NMDA receptor is reported in the pancreatic β-cell. Activation of NMDA receptor reduces the glucose-stimulated insulin secretion. Likewise, NMDA receptor knockout in mouse islets increases glucose-stimulated insulin secretion. NMDA activation in β-cells also promotes cell death under stress. In microphages, activation of NMDA receptor induces ABCA1 degradation which promotes cholesterol accumulation and foam cell formation (75). In liver, NMDA receptor is present on the surface of Kupffer cells, and its activation has been reported to limit inflammasome activation (76).

KYNA acts as a low affinity competitive antagonist of AMPA (α-amino-3-hydroxy-5-methyl-4-isoxazole propionic acid) receptors. It directly acts on the glutamate binding domain. Meanwhile, low concentrations (0.03–30μM) of KYNA potentiate AMPA receptor responses (77). Therefore, KYNA has a dual action on AMPA receptor responses.

KYNA also directly interacts with the glutamate-binding domain of kainate receptors. Its antagonistic effect on this type of receptor is the least potent (IC50 500µM) among the 3 types of glutamate receptors (78).

However, serum KYNA level hardly reaches to micromolar levels. It is unclear whether KYNA in serum or in the periphery is sufficient to antagonize these glutamate receptors.

### Other High Affinity Receptors

The G-protein-coupled receptor 35 (GPR35) is an orphan receptor that was identified in 1998 (79). It was originally described as a receptor for zaprinast, a phosphodiesterase (PDE) inhibitor. Recently, the KYNA was identified as an endogenous ligand for GPR35 with an EC_50_ of 39µM in human and 7.9µM in rat (80). Although the plasmatic concentration of KYNA is often in the nanomolar range in humans, it can become micromolar under inflammatory conditions.

GPR35 is associated with Gi/G0 and G13 proteins (81). Thus, activation of GPR35 reduces the activity of adenylate cyclase (Gi/G0) and/or increases that of the RhoA pathway (G13). GPR35 is expressed in central nervous system and in many peripheral tissues. In humans, significant expression of GPR35 has been detected in the colon, pancreas, small intestine, spleen and immune cells (monocytes, neutrophils, T cells and dendritic cells). The level of its expression is lower in the stomach, skeletal muscle, adipose tissue, pancreatic islets, kidney, liver, and thymus (82). Activation of GPR35 by KYNA has anti-inflammatory effect (83) by inducing autophagy-dependent degradation of NLRP3 in macrophage (84). It also plays anti-nociceptive (85) and anti-asthmatic (86) roles. Furthermore, KYNA enhances Pgc-1α1 and UCP1 expression GPR35 signaling in adipocytes, which suggests KYNA is a signaling molecule which directly controls energy homeostasis (68).

More recently, KYNA has been discovered to significantly increase AMP-activated protein kinase (AMPK) phosphorylation and to ameliorate palmitate-induced inflammation and insulin resistance. It potentially alleviates inflammation and insulin resistance in skeletal muscle and adipose tissues through GPR35/AMPK and SIRT6-mediated pathways (87). It may also ameliorate hepatic steatosis *via* the AMPK/autophagy‐ and AMPK/ORP150‐mediated suppression of endoplasmic reticulum stress (6).

KYNA is also identified as an endogenous ligand for Aryl hydrocarbon receptor (AhR). AhR was originally described as a xenobiotic receptor, also known as the dioxin receptor. It is activated by exogenous ligands, such as flavonoids, natural plant polyphenols, indoles and dioxins. AhR plays multiple roles in xenobiotic metabolism, the regulation of inflammation, development, and the homeostasis of several organs (88). DiNatale and colleagues (89) showed that KYNA is a potent endogenous agonist of AHR with an EC_25_ around 100nM. Activation of AHR by KYNA may lead to IL6 expression in tumor cells.

Moreover, studies have shown that KYNA can also act at nicotinic receptors as a potent noncompetitive antagonist, particularly at the α7 subunit of the nicotinic receptor (90). KYNA inhibits CHRNA7 in a non-competitive manner at physiological concentrations. CHRNA7 was found to be expressed in glutamatergic axon terminals. Activation of CHRNA7 enhances glutamate release. Thus, KYNA may also be involved in the repression of glutamate release at the presynaptic level. This represents another mechanism by which KYNA exerts its anti-glutamatergic effect (90). The characteristics of KYNA receptors are summarized in **Table 2**.

**Table 2:** The characteristic of KYNA receptor.

Receptor	KYNA	Affinity	References
NMDA	antagonist	IC_50_ 7.9-20µM	(70, 91)
Kainate	antagonist	IC_50_ 500µM	(78)
CHRNA7	antagonist	IC_50_ 7µM	(90)
GPR35	agonist	EC_50_ 8-40µM	(80)
AHR	agonist	EC_25_ 100nM	(89)

### Scavenger of Free Radicals

In addition to its receptor-dependent effects, KYNA at high concentrations (100-300 µM) also acts as a potent endogenous antioxidant, as it is a scavenger of free radicals such as hydroxyl radicals (OH▪), superoxide anion (O_2_^-^) and peroxynitrite (ONOO^-^) (92). Since oxidative stress is also critical for the pathogenesis of metabolic diseases (93), the antioxidative properties of KYNA represent an important mechanism in preventing the onset of metabolic diseases.

Interestingly, another study showed that KYNA is not a guaranteed protector against oxidative stress. It exhibits a strong pro-oxidative effect combined with δ-aminolaevulinic acid (ALA), an endogenous precursor of heme and source of hydroxyl radical, and elevates deoxyribose deterioration by 9 times compared to ALA alone (94).

### Mitochondrial Homeostasis

The mitochondrial localization of KYAT suggests a direct release of KYNA into the mitochondria. KYNA plays a key role in the redox balance in the mitochondria. The expression and function of KYATs has been shown to be diminished in rat model with mitochondrial dysfunction (95, 96).

Firstly, the formation of KYNA diverts the pathway from *de novo* synthesis of NAD+/NADH, which regulates the mitochondrial TCA cycle, oxidative state and mitochondrial dynamics, suggesting the involvement of KYNA in the mitochondria energy metabolism regulation. MPTP and 3-nitropropionic acid (3-NA) have inhibitory effects on mitochondrial respiratory chain complexes and on KYAT1 and KYAT2, thus compromise the ATP and KYNA production in the mitochondria (96). Experimentally, FK506, a neuroimmunophilin drug, not only enhanced the formation of KYNA, but abolished the inhibition of KYNA synthesis caused by MPTP and 3-NA. This result suggested that the restoration of respiratory chain function may activate the KYNA synthesis pathway (97). In the case of monogenic form of Leigh Syndrome, the loss-of-function mutation in LRPPRC causes mitochondrial RNA metabolism disorder. The metabolic signature demonstrated a decrease in kynurenine, the precursor of KYNA (98). In patients with Schizophrenia, the prescription of N-acetylcysteine has shown inhibitory effect on KYAT, decreasing the deleterious effect of elevated KYNA on glutamate and dopamine signaling (99). Furthermore, the plasmatic KYNA is positively correlated with fatty acid oxidation and mitochondrial proliferation in the liver of rat (100).

Secondly, KYNA has shown scavenging property of OH▪, O_2_^-^, ONOO^-^ (92, 101). In a preparation of oocytes, KYNA significantly reduced ROS and lipid peroxidation induced by FeSO_4_. For 3-Methylglutaric acid (3MGA) which accumulates in the brains of children coursing with metabolic acidurias, experiments showed that 3MGA induced an increase in ROS production and lipid peroxidation and a decrease in mitochondrial function. Addition of KYNA showed antagonist effects (102).

Thirdly, as has been discussed in 4.1, KYNA has potent antagonist effects over NMDAR (103), which may reduce de notorious effect of excitotoxicity on mitochondria, *via* decreasing excessive intracellular Ca^2+^ as example (104).

Finally, KYNA was shown to directly impair respiratory parameters of heart mitochondria. Moreover, the effect is selective for complex I (105, 106). However, this respiratory chain-modulating property was only observed in heart mitochondria, and is absent for brain and liver mitochondria, suggesting profound differences between tissular mitochondria content and helping to explain the tissue-specific effect of KYNA. It should be pointed out that these *in vivo* experiments were carried out with high concentration of KYNA (125-1000 μM) which is rarely achieved under physiological conditions (92).

## Conclusion

Increasing evidence indicates that KYNA can act as a signaling molecule to regulate energy expenditure in a network integrating nutrition, physical exercise, inflammation and metabolic diseases besides its neuro-protector role in the central nervous system (**Table 3**). Targeting KYNA signaling network or its metabolic pathway harbors high potentials to expand the range of strategy to prevent and treat metabolic diseases.

**Table 3:** The roles of KYNA in metabolic diseases.

Organ/cell	KYNA production	Effects	Associated metabolic disease
Liver	High expression levels of TDO/IDO/KYATs were detected in liver (12)	Activation of GPR35 inhibits the development of NAFLD (5)	NAFLD
		Inhibition of Kuffer cells NMDA receptor by KYNA limits inflammasome activation (76)	Metabolic inflammation
		Activation of AMPK/autophagy‐ and AMPK/ORP150 pathway by KYNA ameliorate endoplasmic reticulum stress and hepatic steatosis (6)	Hepatic steatosis
Adipose tissue/adipocyte	Expression of IDO1/KYAT1/KYAT3 were detected in adipocytes (3)	Activation of GPR35 by KYNA promotes the expression of PGC1-α and UCP1 (67)	Insulin resistance
		Activation of GPR35/AMPK and SIRT6 pathways by KYNA reduces inflammation and insulin resistance in adipocytes (87)	Metabolic inflammation
Muscle	Endurance essences enhance KYATs expression and promote KYNA production (64, 65, 67)	Activation of GPR35/AMPK and SIRT6 pathways by KYNA reduces inflammation and insulin resistance in skeletal muscle (87)	Insulin resistance Metabolic inflammation
Immune cell	KYAT1/KYAT2 expressions were detected in in both unstimulated and stimulated macrophage (107)	Antagonize NMDA receptor by KYNA inhibits ABCA1 degradation (75)	Cholesterol accumulation
		Activation of GPR35 by KYNA induces autophagy-dependent degradation of NLRP3 in macrophage (84)	Metabolic inflammation
Pancreas	KYNA were detected in pancreas fluid, expression of TDO/KYATs were detected in pancreatic islets (9, 13)	High concentration of KYNA enhances glucose stimulated insulin secretion (13)	Type 2 diabetes
Mammary gland	KYNA content in breast milk gradually increases in different lactation periods (10)	KYNA may act as an anti-obese agent for children (10)	Obesity

## Author Contributions

ZS designed and reviewed the article. DZ wrote the draft. XDZ revised the content. JL collected references. All authors contributed to the article and approved the submitted version.

## Funding

This research was funded by the Henan Provincial Post-doctorate Research Fund and ENN Research Fund.

## Conflict of Interest

The authors declare that the research was conducted in the absence of any commercial or financial relationships that could be construed as a potential conflict of interest.

## Publisher’s Note

All claims expressed in this article are solely those of the authors and do not necessarily represent those of their affiliated organizations, or those of the publisher, the editors and the reviewers. Any product that may be evaluated in this article, or claim that may be made by its manufacturer, is not guaranteed or endorsed by the publisher.
